# Real‐world outcomes of patients with resected stage III melanoma treated with adjuvant therapies

**DOI:** 10.1002/cam4.7257

**Published:** 2024-06-20

**Authors:** Danai Dima, Nerea Lopetegui‐Lia, Olisaemeka Ogbue, Bennett Osantowski, Fauzia Ullah, Xuefei Jia, Jung Min Song, Brian Gastman, James Isaacs, Lucy Boyce Kennedy, Pauline Funchain

**Affiliations:** ^1^ Department of Hematology‐Oncology Taussig Cancer Institute, Cleveland Clinic Foundation Cleveland Ohio USA; ^2^ Department of Internal Medicine Cleveland Clinic Foundation Cleveland Ohio USA; ^3^ Department of Biostatistics Cleveland Clinic Foundation Cleveland Ohio USA; ^4^ Department of Plastic Surgery Cleveland Clinic Foundation Cleveland Ohio USA; ^5^ Division of Oncology, Stanford Cancer Institute Stanford University School of Medicine Stanford California USA

**Keywords:** adjuvant therapy, adverse events, immunotherapy, stage III melanoma, targeted therapy

## Abstract

**Background:**

Both immunotherapy (IO) and targeted therapy (TT) are used as adjuvant (adj) treatment for stage III melanoma, however, data describing real‐world outcomes are limited. In addition, a significant proportion of patients relapse, for whom best management is unclear. The aim of our study was to assess the efficacy, and safety of adj anti‐PD1 IO and TT in a real‐world cohort of patients with resected stage III melanoma, and further delineate patterns of recurrence and treatment strategies.

**Methods:**

We retrospectively analyzed 130 patients who received adj therapy (100 anti‐PD1 IO and 30 TT).

**Results:**

At a median follow‐up of 30 months, median relapse‐free survival (RFS) was 24.6 (95% CI, 17–not reached [NR]) versus 64 (95% CI, 29.5–NR) months for the TT and IO groups, respectively (*p* = 0.26). Median overall survival (OS) was NR for either subgroup. At data cutoff, 77% and 82% of patients in TT and IO arms were alive. A higher number of grade ≥3 treatment‐related adverse events (AEs) were noted in the IO group (11% vs. 3%), however, a higher proportion of patients permanently discontinued adj therapy in the TT group (43% vs. 11%) due to toxicity. Strategies at relapse and outcomes were variable based on location and timing of recurrence. A significant number of patients who relapsed after adj IO received a second round of IO. Among them, patients who were off adj IO at relapse had superior second median RFS (mRFS2), compared to those who relapsed while on adj IO; mRFS2 was NR versus 5.1 months (95% CI, 2.5–NR), respectively, *p* = 0.02.

**Conclusion:**

In summary, both TT and IO yielded prolonged RFS in a real‐world setting, however, longer follow‐up is needed to determine any potential OS benefit. Adj therapy, particularly TT, may not be as well tolerated as suggested in clinical trials, with lower completion rates (59% vs. 74%) in a real‐life setting. Overall, patients who relapse during adj therapy have poor outcomes, while patients who relapse after discontinuation of adj IO therapy appear to benefit from IO re‐treatment.

## Graphical abstract

Both immunotherapy and targeted therapy are used as adjuvant treatment modalities for stage III melanoma. However, data describing real‐world outcomes are limited. Our study aimed to assess the efficacy and safety of adjuvant anti‐PD‐1 immunotherapy and targeted therapy in a real‐world cohort of 130 patients with resected stage III melanoma, and further delineate patterns of recurrence and treatment strategies.

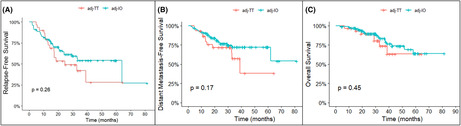

## 1 INTRODUCTION

Over the last decade, significant drug advancements have radically shifted the treatment paradigm of melanoma leading to improved patient outcomes.^1^, ^2^, ^3^ Immunotherapy (IO) with anti‐CTLA4 and anti‐PD1 monoclonal antibodies, as well as targeted therapy (TT) with BRAF/MEK inhibitors, demonstrated improved survival of patients with metastatic disease.^4^, ^5^, ^6^, ^7^, ^8^ More recently, these treatments have been studied in the adjuvant (adj) setting for resected stage III/IV melanoma, also showing clinical benefit.

Results from three major trials of adj anti‐PD1 therapy provided concrete evidence of significant decrease in disease recurrence with the use of pembrolizumab (pembro) and nivolumab (nivo), leading to their approval in the adj setting.^9^, ^10^, ^11^, ^12^ Similarly, TT (dabrafenib–trametinib) was also proven to decrease risk for relapse, and hence, was approved for BRAF‐mutant disease.^13^ Notably, while there is some evidence to suggest overall survival (OS) benefit with TT, prospective data demonstrating OS advantage with adj anti‐PD1 IO is lacking. Improved OS was seen with adj anti‐CTLA4 IO (ipilimumab [ipi]) compared to high dose interferon in one trial.^14^, ^15^ However, further prospective studies showed that adj ipi induced inferior relapse‐free survival (RFS) rates compared to adj nivo, while the combination of adj ipi/nivo was not superior to nivo alone^16^, ^17^; thus, single‐agent ipi is not routinely used in the adj setting. Interestingly, a large‐scale analysis of the National Cancer Database showed that adj IO improved OS when compared to surveillance alone but significant benefit was only seen in patients with resected stage IIIC disease.^18^

Despite the significant impact of adj therapy seen in pivotal clinical trials, limited data exist regarding outcomes in real‐world populations. In addition, limited data are available regarding nature and patterns of recurrence despite adj therapy, as well as the management and outcomes of patients who experience progression.^19^, ^20^, ^21^, ^22^ Furthermore, the tolerability of either adj modality in a real‐life setting or the ability of patients to complete the planned course, especially those with Eastern Cooperative Oncology Group (ECOG) performance status (PS) >1, are not well characterized. The aim of our study was to address the aforementioned gaps in literature, by assessing the efficacy and safety of adj anti‐PD1 and TT in a real‐world, single‐center cohort of patients with resected stage III melanoma, and further describe patterns of recurrence and treatment strategies.

## 2 METHODS

This retrospective study included adult patients with resected stage III melanoma according to the AJCC 8th edition staging system who received adj therapy with either anti‐PD1 or TT between January 1, 2017 and January 7, 2023.^23^ Patients with other advanced or active malignancies were excluded. A total of 130 patients were identified from the Cleveland Clinic (CC) Solid Tumor Registry. Data were extracted from the electronic medical record by trained abstractors. The CC Institutional Review Board approved the study, which was granted a waiver of consent.

Efficacy, survival and safety outcomes were analyzed for patients who received at least one dose of adj treatment. RFS was defined as the time from adj therapy initiation until first evidence of disease progression or death from any cause, whichever occurred first; distant metastasis‐free survival (DMFS) was defined as the time from adj therapy initiation to the development of first distant metastasis or death from any cause, whichever occurred first. OS was defined as the time between date of adj therapy initiation and the date of death from any cause. Non‐evaluable patients for response assessment to systemic therapy at first relapse were defined as patients who had locoregional relapse and underwent successful surgical resection of their tumor prior to systemic therapy initiation.

Statistical analysis was done with the use of Fisher's Exact test and Kaplan–Meier method for RFS, DMFS, and OS estimations. Log‐rank tests were applied for comparisons among the subgroups. Results were considered statistically significant if the two‐sided *p*‐value was less than or equal to 0.05. R software (version 3.6.2) was used for statistical analysis.

## 3 RESULTS

### 3.1 Patient characteristics

This study included 130 patients; baseline characteristics are summarized in Table 1. Both IO and TT groups had comparable characteristics. The median age at adj therapy initiation was 58 (range 25–84) years. All patients except one were diagnosed with cutaneous melanoma. Thirteen percent of patients were diagnosed with stage IIIA, 40% with stage IIIB, 43% with stage IIIC, and 4% with stage IIID disease. Of the included patients, 100 (77%) received anti‐PD1 (89% nivo, 11% pembro), and 30 (23%) received TT (100% dabrafenib–trametinib). The median duration of adj TT and IO therapy was 10.5 and 11 months, respectively. At data cutoff, among the 30 patients who received TT, 14 (47%) completed 1 year of planned treatment, whereas among the 100 patients who received IO, 67 (67%) completed 1 year of planned treatment. Thirteen patients (43%) in the TT group and 11 (11%) in the IO group discontinued adj therapy prematurely due to treatment‐related adverse events (AEs) (Table 2).

**TABLE 1:** Baseline characteristics of patients receiving adjuvant therapy.

Clinical characteristics	Study population	Targeted therapy	Immunotherapy
	*N* = 130 (100%)	*N* = 30 (100%)	*N* = 100 (100%)
Age, median (range)	58 (25–84)	54 (33–80)	59 (25–84)
Male gender—*n* (%)	78 (60)	15 (50)	63 (63)
White race—*n* (%)	130 (100)	30 (100)	100 (100)
ECOG PS, median (range)	0 (0–3)	0 (0–2)	0 (0–3)
Stage III—*n* (%)			
A	17 (13)	5 (17)	12 (12)
B	52 (40)	10 (33)	42 (42)
C	56 (43)	14 (47)	42 (42)
D	5 (4%)	1 (3)	4 (4)
Primary site—*n* (%)			
Head and Neck	29 (22)	6 (20)	23 (23)
Trunk	34 (26)	11 (37)	23 (23)
Lower extremities	30 (23)	5 (17)	25 (25)
Upper extremities	37 (29)	8 (26)	29 (29)
BRAF mutation—*n* (%)			
Positive	72 (56)	30 (100)	42 (42)
Negative	51 (39)	0 (0)	51 (51)
Not documented	7 (5)	0 (0)	7 (7)
KIT mutation—*n* (%)			
Positive	5 (4)	0 (0)	5 (5)
Negative	83 (64)	20 (67)	63 (63)
Not documented	42 (32)	10 (33)	32 (32)
NRAS mutation—*n* (%)			
Positive	11 (8)	0 (0)	11 (11)
Negative	79 (61)	20 (67)	59 (59)
Not documented	40 (31)	10 (33)	30 (30)
Type of lymph node involvement (for stage ≥ IIIb disease, *n* = 61)—*n* (%)			
Macroscopic	2 (3)	0 (0)	2 (4)
Microscopic	59 (96)	15 (100)	44 (96)

**TABLE 2:** Duration of adjuvant therapy, timing and patterns of relapse.

	Targeted therapy	Immunotherapy
	*N* = 30	*N* = 100
Duration of adjuvant therapy—*n* (%)		
Completed 1 year of planned adj Rx	14 (47)	67 (67)
Discontinued prematurely due to PD	3 (10)	20 (20)
Discontinued prematurely due to AEs	13 (43)	11 (11)
Loss to follow‐up	0 (0)	2 (2)
Timing of recurrence—*n* (%)		
Total patients who relapsed	16 (53)	38 (38)
Relapse ON adjuvant therapy	4 (13)	20 (20)
Relapse OFF adjuvant therapy	12 (40)	18 (18)
Had completed adj Rx	5 (42)	13 (72)
Had discontinued due to AEs	7 (58)	5 (28)
Location of recurrence—*n* (%)	*N* = 16	*N* = 38
Locoregional relapse	6 (38)	19 (50)
Distant relapse	10 (62)	19 (50)
Brain	4 (25)	5 (13)
Lung	4 (25)	7 (18)
Liver	2 (12.5)	4 (11)
Spleen	1 (6)	1 (3)
Bone	1 (6)	5 (13)
Peritoneum	0 (0)	1 (3)
Distant lymph nodes	0 (0)	8 (21)
Soft tissue	0 (0)	4 (11)
Stomach	0 (0)	1 (3)
Small bowel/colon	0 (0)	2 (5)
Pancreas	0 (0)	1 (3)
Multiorgan	1 (6)	10 (26)

Abbreviations: Adj, adjuvant; AEs, adverse events; PD, disease progression; Rx, therapy.

### 3.2 Duration and efficacy of adjuvant therapy

After a median follow‐up of 30 months, 54 (42%) out of the 130 patients relapsed: 16 (53%) from the TT group and 38 (38%) from the IO group. In the IO group, similar proportions of patients relapsed while on and off adj therapy (20% vs. 18%), whereas in the TT group, most patients relapsed while off therapy (40% vs. 13%). Of those who relapsed off adj therapy, 42% and 72% had previously completed the 1‐year planned course of adj TT and IO, respectively; the remaining had ceased therapy prematurely due to treatment‐related AEs. The occurrence of relapse in patients who completed adj therapy versus those who did not (excluding patients who relapsed while on adj) per treatment modality was 38% versus 54%, *p* = 0.73 for the TT group, and 19% versus 45%, *p* = 0.17 for the IO group.

**FIGURE 1:**
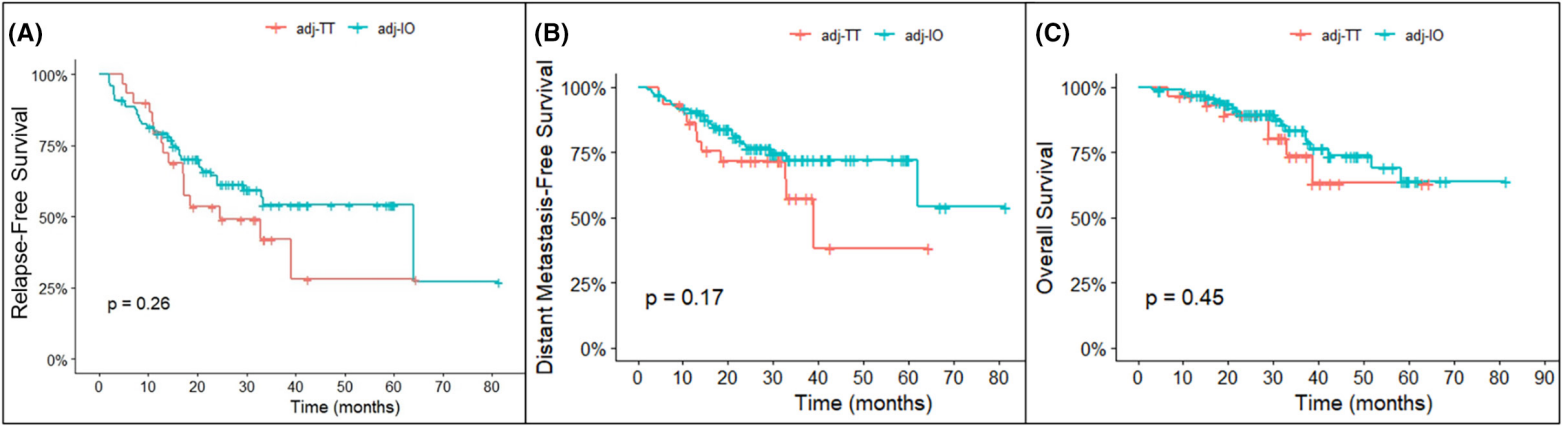
Kaplan–Meier curves of (A) relapse‐free survival (B) distant metastasis‐free survival, and (C) overall survival of patients treated with adjuvant (adj) targeted therapy (TT) versus adjuvant immunotherapy (IO).

The estimated 12‐month, 18‐month, and 3‐year RFS rates for the adj TT group were 79.6% (95% CI, 66.3–95.6), 57.3% (95% CI, 41.5–79.1), and 42% (95% CI, 25.7–68.8), respectively, whereas for the adj IO group were 79.3% (95% CI, 71.6–87.8), 70.2% (95% CI, 61.4–80.1), and 54.1% (95% CI, 43.3–67.6), respectively. The estimated median RFS was 24.6 months (95% CI, 17–not reached [NR]) for the adj TT group versus 64 months (95% CI, 29.5–NR) for the adj IO group, *p* = 0.26 (Figure 1A). Likewise, the estimated 12‐month, 18‐month, and 3‐year DMFS rates for the adj TT group were 79.6% (95% CI, 66.3–95.6), 57.3% (95% CI, 41.5–79.1), and 42% (95% CI, 25.7–68.8), respectively, whereas for the adj IO group were 79.3% (95% CI, 71.6–87.8), 70.2% (95% CI, 61.4–80.1), and 54.1% (95% CI, 43.3–67.6), respectively. The estimated median DMFS was 39 months (95% CI, 32.8–NR) for the TT group versus NR (95% CI, 62–NR) for the IO group, *p* = 0.17 (Figure 1B).

For the subgroup of the BRAF‐mutated patients only, the estimated median RFS was 24.6 months (95% CI, 17–NR) with adj TT versus 29.5 months (95% CI, 16.7–NR) with adj IO, *p* = 0.73; whereas the estimated median DMFS was 39 (95% CI, 32.8–NR) with adj TT, versus 62 (95% CI, NR–NR) months with adj IO, *p* = 0.62 (Table S1; Figure S1).

### 3.3 Safety and tolerability of adjuvant therapy

A considerable number of patients in each group developed treatment‐related AEs: 16 (53%) in the TT group and 69 (69%) in the IO group. Most common AEs are shown in Table 3. IO had a wider variety and a higher incidence of grade 3–4 AEs in contrast to TT, where no grade 4 AEs were seen, and just one patient developed grade 3 panuveitis. However, a higher proportion of patients in the TT group permanently discontinued adj therapy due to AEs compared to the IO group: 43% versus 11%, respectively, *p* = 0.005.

**TABLE 3:** Most common treatment‐related adverse events.

Adverse event	Targeted therapy		Immunotherapy	
	Any grade	Grade 3–4	Any grade	Grade 3–4
Fevers	8	0	–	–
Fatigue	5	0	19	0
Headache	3	0	1	0
Tinnitus	–	–	1	0
Neuropathy	–	–	2	0
Gastrointestinal				
Transaminitis	2	0	7	2
Hepatitis	–	–	1	1
Nausea/GI disturbance	5	0	3	0
Diarrhea/colitis	3	0	8	4
Pneumonitis	–	–	5	2
Arthritis/arthralgias	1	0	11	0
Generalized muscle pain	2	0	3	0
Uveitis/eye toxicity	4	1	1	0
Dermatologic				
Rash	3	0	10	1
Folliculitis	–	–	1	0
Vitiligo	–	–	1	0
Dry skin/mouth	–	–	2	0
Hair thinning	–	–	1	0
Endocrine				
Hypophysitis, adrenal insufficiency	–	–	1	1
Thyroiditis/hypothyroidism	–	–	11	0
Existing autoimmune disease flare	–	–	2	1
Autoimmune pancreatitis leading to DM	–	–	1	1
Triple M syndrome	–	–	1	1
Infusion related reaction	–	–	2	0
Number of patients experiencing AEs—*N* (%)	16 (53)	1 (3)	69 (69)	11 (11)

Abbreviations: DM, diabetes mellitus; triple M syndrome, myositis, myocarditis, myasthenia gravis.

Among the IO group, one patient developed autoimmune pancreatitis leading to type 1 diabetes mellitus (grade 4 AE). The most common grade 3 AEs were: transaminitis/hepatitis, diarrhea/colitis, triple M syndrome (myositis, myocarditis, myasthenia gravis), and endocrinopathies, including hypophysitis and adrenal insufficiency. Triple M syndrome was debilitating leading to ECOG PS of 4. With IO, six patients were admitted to the hospital for management of immune‐related AEs (irAEs). The patient who developed triple M syndrome required a total of five hospital admissions, and was treated with corticosteroids, intravenous immunoglobulins, therapeutic plasma exchange, and abatacept. The rest of the patients with serious irAEs were admitted for pneumonitis, colitis, arthritis, and thyroid disorder, and received routine management, including corticosteroids, biologics and hormone replacement, as appropriate. Given previous observations that irAEs typically portend better IO outcomes,^24^ we examined RFS among patients with none, grade 1–2 and grade 3–4 irAEs, and found no significant difference among the subgroups (*p* = 0.55). Among patients who received TT, only one patient required hospital admission due to acute treatment‐related AEs. The patient had fevers, nausea, vomiting, diarrhea, and acute kidney injury/interstitial nephritis that ultimately led to discontinuation of TT, and was treated with supportive care.

Ten patients (8%) had ECOG PS more than one at adj therapy initiation. Of these, three received adj TT, while the remaining seven received adj IO. All patients had an ECOG PS score of 2, except for one patient in the IO group who had an ECOG PS score of 3. None of these patients permanently discontinued adj therapy due to treatment‐related AEs. Three patients with ECOG PS 2 experienced grade 2 colitis, grade 2 thyroiditis, and grade 1 pneumonitis related to IO, respectively; the rest did not have any AEs.

### 3.4 Patterns of recurrence and management

In the TT group, 38% of relapses were locoregional and 62% were distant; in the IO group, 50% of relapses were locoregional and 50% distant. Details regarding timing of recurrence and locations of distant relapse are summarized in Table 2.

**FIGURE 2:**
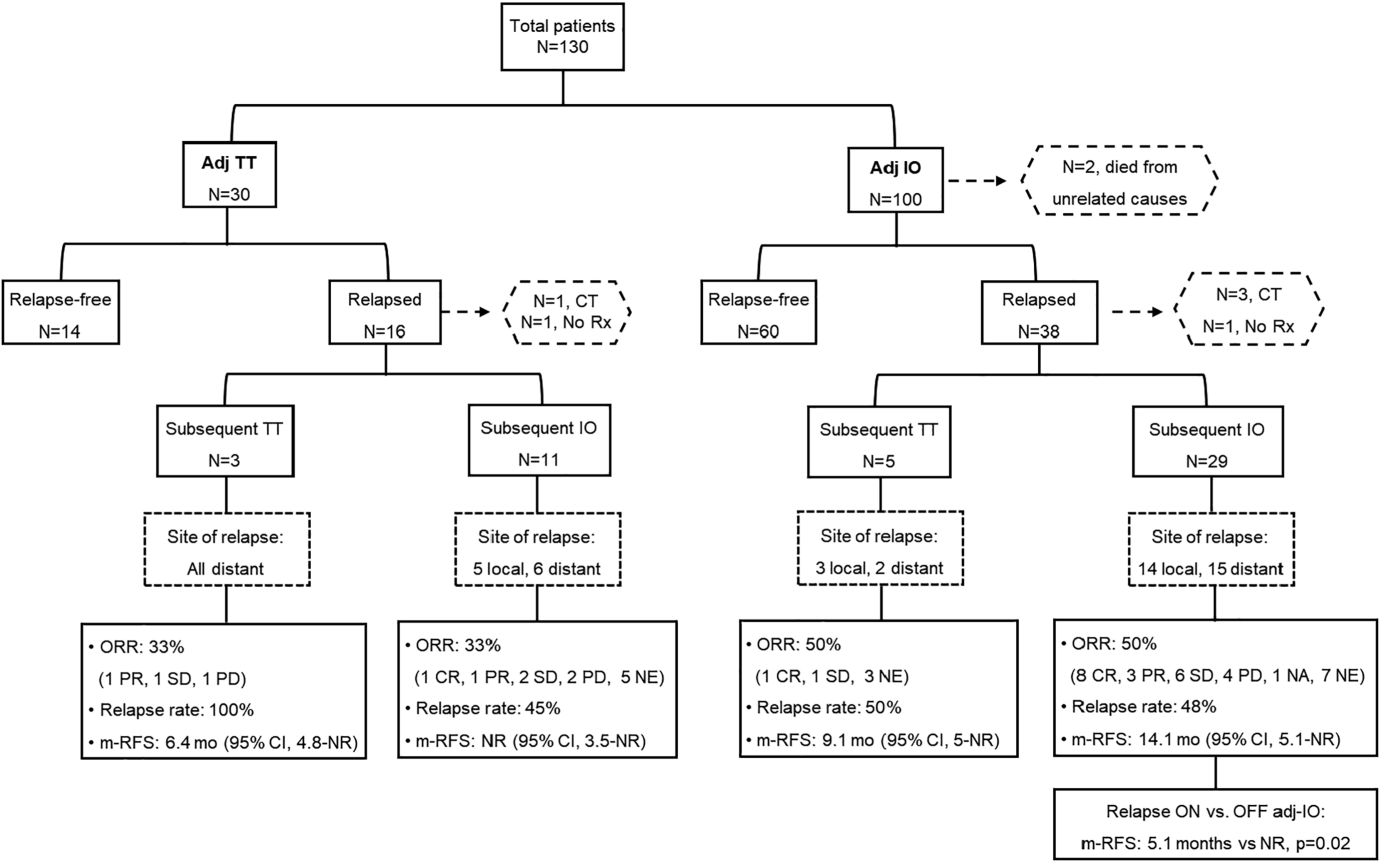
Flow diagram of study patients, including response rates, relapse rates, and median relapse‐free survival based on type of subsequent systemic therapy received at first relapse post adjuvant treatment. Adj, adjuvant; CR, complete response; CT, clinical trial; IO, immunotherapy; m‐RFS, median relapse‐free survival; mo, months; NA, not assessed; NE, non‐evaluable; NR, not reached; PD, progressive disease; PR, partial response; Rx, treatment; SD, stable disease; TT, targeted therapy.

**FIGURE 3:**
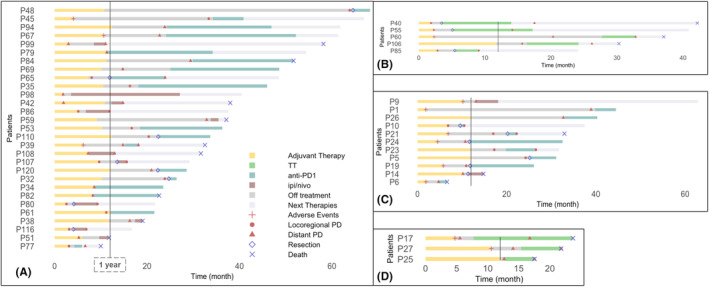
Swimmer plot for relapsed patients: duration of adjuvant therapy, patterns of recurrence, type of therapy at first recurrence, and survival outcomes. (A) adjuvant IO➔ IO at first relapse, (B) adjuvant IO➔ TT at first relapse, (C) adjuvant TT➔ IO at first relapse, (D) adjuvant TT➔ TT at first relapse.

The management of 53 patients who relapsed is outlined in Figures 2 and 3. For the adj TT group, 5/6 patients with locoregional progression underwent resection of their tumor. In addition, 5/6 patients started anti‐PD1 monotherapy, and the remaining one was enrolled in a clinical trial. Of the patients who received anti‐PD1 therapy, three further progressed again with locoregional disease. Among the 10 patients who progressed with distant metastasis, one died shortly thereafter due to multiorgan progression including brain lesion, four patients received nivo, two ipi/nivo, and three TT combination. Overall response rates were 50%, 0%, and 33%, respectively, for the aforementioned groups who received subsequent treatment. Detailed responses to systemic therapy and further progression are summarized in Table S2.

Among the 19 patients in the adj IO group who relapsed locoregionally, 95% underwent local intervention, and 95% received systemic therapy. Local treatments included: 56% tumor resection, 33% intralesional talimogene laherparepvec (TVEC), and 11% intralesional investigational therapies in the setting of clinical trial. Systemic therapies included: 78% IO (61% anti‐PD1, 17% ipi/nivo), 17% TT, and 5% experimental therapies. Of the 14 patients who received a second round of IO, 50% further progressed including all patients who received ipi/nivo. Likewise, all patients who received subsequent TT also experienced further disease progression. Detailed combinations of local interventions with systemic agents and outcomes are shown in Table S3.

Among the 19 patients in the adj IO group who relapsed with distant metastasis, 15 (79%) were retreated with IO, 2 (11%) received subsequent TT, 1 (5%) was enrolled in a clinical trial, and 1 (5%) died shortly thereafter without receiving any further therapy. Of the 15 patients who were retreated with IO, eight (53%) received anti‐PD1 therapy, six (40%) ipi/nivo, and one (7%) ipi. Overall response rate of re‐treatment with IO was 40%. For the different types of IO response rates were: anti‐PD1 therapy 63%, ipi/nivo 17%, and ipi 0%. Eventually, 5/15 patients further progressed, of whom none had previously achieved any response. For the two patients who received subsequent TT, one achieved complete response, and the other stable disease. Detailed responses to systemic therapy and further progression are outlined in Table S4.

The best overall response to systemic therapy at first relapse based on timing of relapse (on vs. off adjuvant therapy) and location of relapse (locoregional vs. distant) for the entire study population are summarized in Tables S5 and S6.

### 3.5 Relapse‐free survival‐2 and overall survival outcomes

**FIGURE 4:**
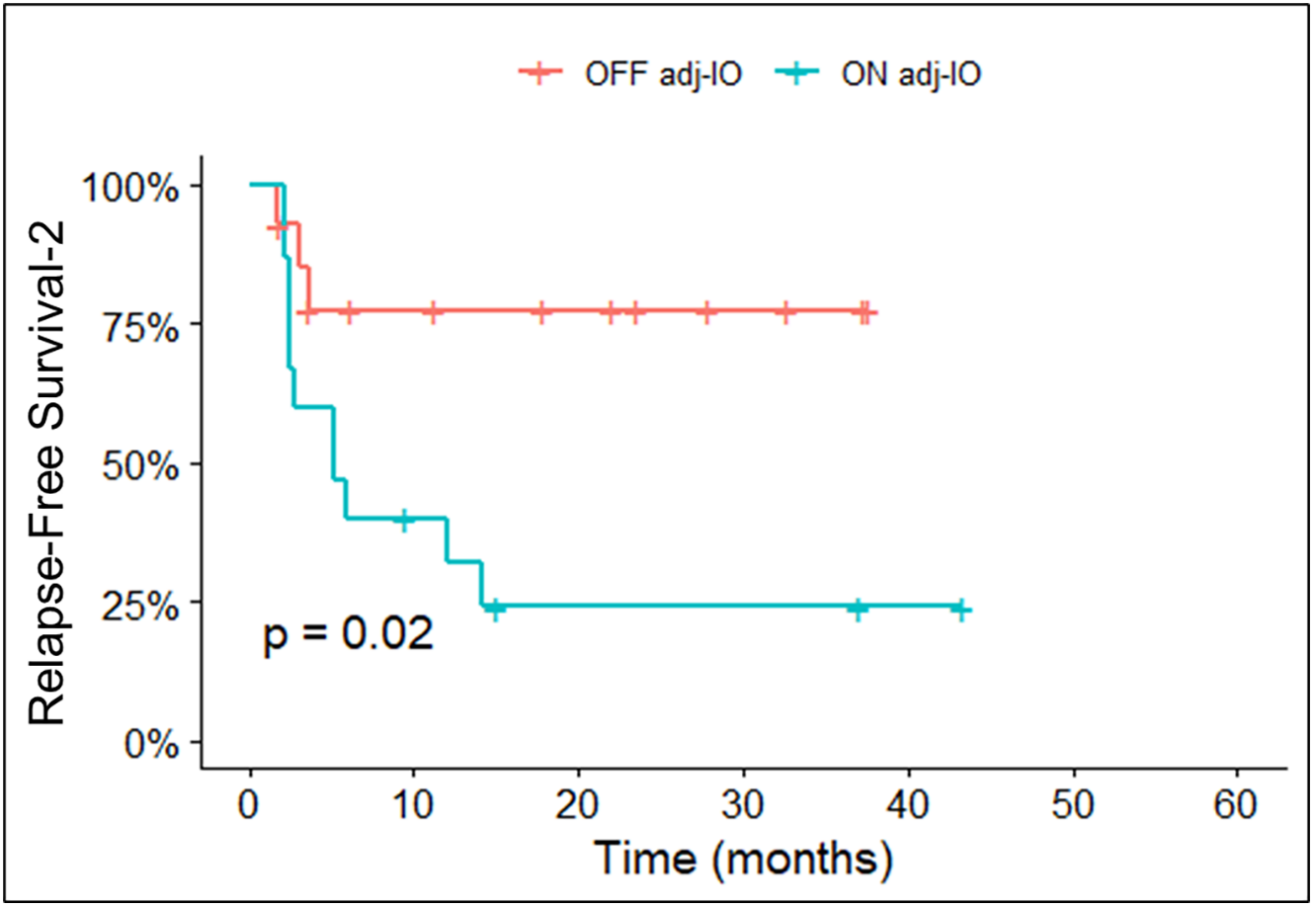
Kaplan–Meier curve of second relapse‐free survival with immunotherapy re‐treatment (RFS‐2) between patients who relapsed ON adjuvant IO versus patients who relapsed OFF adjuvant IO.

A total of 29 patients were re‐treated with IO at first relapse. Of these, 11 (38%) had previously completed 1 year of adj IO, 15 (52%) had relapsed while on adj IO, and 3 (10%) had prematurely ceased adj IO due to toxicity. For the patients who relapsed while on adj IO, a second course of IO was immediately started. For patients who had completed 1 year of adj IO or had previously discontinued IO due to AEs, median time between last dose of adj IO and first dose of IO re‐treatment was 12.5 months (range 7–30). Patients who were off adj IO at relapse had superior second median RFS to re‐treatment with IO (mRFS2), compared to those who experienced first recurrence while on adj IO; mRFS2 was NR versus 5.1 months (95%CI, 2.5–NR), *p* = 0.02, respectively (Figure 4).

At data cutoff, a total of 25 patients had died. The majority (84%) died from disease progression, while four patients (in the adj IO group) died from other causes (sepsis, myocardial infarction, pneumonia, and neuromuscular disease). The median OS was not reached for either the adj TT or IO groups; 4‐year OS rate was 63.2% (95% CI, 42.5–94.1) and 73.7% (95% CI, 62.6–86.8), respectively (Figure 1C). At the end of the study, 77% and 82% of patients in the adj TT and IO groups, respectively, were alive.

## 4 DISCUSSION

In this retrospective study of patients with resected stage III melanoma, we evaluated the efficacy and tolerability of adj therapy, as well as patterns of recurrence, and management following relapse in a real‐world setting. This is important given the lack of head‐to‐head comparisons of adj TT and IO in clinical trials and lack of prospective data guiding therapy selection at the time of recurrence despite adj therapy for patients with initially resected stage III disease.

In our real‐world cohort, the RFS of patients who received adj TT was somewhat lower than the RFS reported in the COMBI‐AD trial, and additionally, we observed a higher rate of TT discontinuation due to toxicity in our study compared to the trial population,^25^ which could partially explain this difference in RFS. On the other hand, real‐world RFS with adj IO appears to agree with that of Checkmate‐238 and Keynote‐054 trials.^9^, ^10^ While our findings showed no statistically significant difference between the RFS of the patients who received adj TT and IO, the median RFS of those who received IO was longer (64 vs. 24.6 months) which might be considered clinically significant. Our findings differ from a recently published study by De Falco et al, who reported that RFS was in favor of the TT group; however, median follow‐up was shorter compared to our study, and the median RFS was NR for both subgroups.^26^ Furthermore, baseline characteristics in our adj TT and IO populations were well balanced, making comparisons overall relevant. Another real‐world study by de Meza et al reported a better 12‐month RFS rate with adj TT than IO, but this benefit was no longer observed at 18 months.^27^ Again, the median RFS was NR and the median follow‐up was only about 1 year. Despite a median follow‐up of 30 months in our study, OS outcomes remain immature; longer follow‐up is necessary to determine any potential benefit in real‐world setting.

Our data showed that AEs of any grade occurred in 69% and 53% of adj IO and TT groups, respectively, which is lower than what has been reported in the registration trials (77.8–85.2% and 97%, respectively).^17^, ^25^, ^28^ Rate of treatment‐related AEs grade ≥3 in the IO group of our study were in line with what was previously reported in Checkmate‐238 and Keynote‐054 trials (11% vs. 14.4–14.7%)^17^, ^28^; however, in the TT group, rate of AEs grade ≥3 was remarkably lower compared to the COMBI‐AD trial (3% vs. 41%).^25^ Similar to our study, real‐world data from the Dutch and Italian groups also reported lower rates of severe AEs (11.5% and 17.2%) with adj TT.^26^, ^27^ However, both in our study and other real‐world reports, including the Dutch study, discontinuation rates of adj TT were higher (41% and 29–39%) than the COMBI‐AD trial (26%) or trials in the metastatic setting.^27^, ^29^ The discordance in low grade ≥3 AEs and high cessation rates due to toxicity is most likely due to less meticulous AE grading, as well as lower tolerance for AEs by both patients and practitioners, both of which are frequently seen in real‐life practice. The high discontinuation rates could highlight that tolerability may be different from trial participants, particularly in the adj space where patients generally maintain normal activities and are less willing to tolerate daily symptoms that may impair quality of life, compared to the metastatic setting.

In contrast, premature discontinuation rates of adj IO in our study (11%) were similar to the Checkmate‐238 and Keynote‐054 trials (7.7%–13%), but slightly lower than other real‐world reports (17.9%–21.5%).^29^, ^30^ Overall, it appears that adj IO is better tolerated, which is in agreement with prior reports of real‐word experiences.^31^ Although numerically more patients finished 1 year of adj IO, the rates of hospitalizations were higher in this group, emphasizing that toxicity can be severe when it does occur. For patients with ECOG PS >1, either of the adj modalities appeared safe and tolerable; none developed grade ≥3 AEs or had to discontinue therapy due to toxicity. This point is especially important given that these patients were excluded from major clinical trials, and safety outcomes for this population have not been well characterized.

In patients with melanoma progression despite adj TT, our results suggest that subsequent IO might offer disease control; however, responses were lower (33%) than previously reported by Bhave et al who found an ORR of 46%–62% to subsequent anti‐PD1 with or without ipi.^22^ This low response rate may be a signal of a similar phenomenon of reduced IO efficacy following TT as was observed in the metastatic setting in the DREAMseq study, although this pattern has not yet been reported as extensively in the adj setting.^8^ Subsequent therapy with TT showed some activity (ORR 33%), however, all patients eventually relapsed and died. Prior studies have described similar response rates, between 25% and 43%, to re‐treatment with TT for both initial stage III and metastatic disease.^22^, ^32^, ^33^, ^34^ Notably, a significant portion of distant relapses included brain involvement that responded poorly to subsequent treatment with either TT or IO leading to dismal survival.

In patients with melanoma progression despite adj IO, systemic therapy appears active, but responses varied by drug class, timing, and location of recurrence. In the most recent report of the Keynote‐054 trial, the overall 5‐year RFS2 rate was 68.2% in the pembro group, where an anti–PD1‐based treatment, anti‐CTLA4 and BRAF/MEK inhibitor combination were administered to 27.2%, 18.4%, and 14.7% of patients at first recurrence, respectively.^35^ In our study, 76% of patients who progressed despite adj IO were retreated with IO (66% anti‐PD1, 31% ipi/nivo, 3% ipi) at first relapse. For those with evaluable disease, ORR was 50% and stable disease rate was 27%. This data are comparable to published studies in advanced melanoma that has failed anti‐PD1 monotherapy, where responses were approximately 30% with use of subsequent anti‐PD1 with or without ipi.^36^, ^37^ Further analysis showed that patients who were off adj IO at first relapse, had superior RFS2 to re‐treatment with IO, compared to those who experienced first recurrence while on adj IO, which aligns with previous findings.^21^ The median time between last dose of adj IO and IO re‐initiation was approximately 1 year (range of 7–30 months), suggesting that a second round of IO can be effective in patients who have been off adj IO, in particular for more than 6 months. The poor outcomes to re‐treatment with IO, including ipi/nivo, of patients who relapsed during adj anti‐PD1 highlights that this subgroup likely needs early consideration of novel therapies including enrollment in clinical trials.

Most patients who relapsed locoregionally underwent local resection or TVEC.^38^ However, this group experienced a significant rate of subsequent second relapse both locally and distantly. Defining the optimal management of patients with locoregional relapse following adj therapy is an unmet need. In this study, although numbers are small, for patients who received adj IO and relapsed with locoregional disease, subsequent TT showed 100% failure rate in preventing further relapse, whereas subsequent IO led to 50% of patients being disease free at data cutoff. A recent retrospective analysis of 55 patients receiving second adj TT for resected stage III/IV disease after failure of adj PD1‐based IO, reported a median RFS of 33.4 months and DMFS of NR.^39^ Most patients recurred after cessation of second adj TT, and the proportions of patients experiencing further local and distant relapses were similar. The role of TVEC in locoregional recurrence post adj therapy is also not well characterized. Herein, six patients who progressed locoregionally received TVEC/anti‐PD1 with an ORR of 67% and 50% having not yet relapsed at data cutoff. This suggests that TVEC/anti‐PD1 combination may be an effective option at locoregional recurrence.

Limitations of our study include its retrospective design, single institutional nature, and limited sample size. Furthermore, the duration of median follow‐up was relatively short, not allowing comparison of long‐term survival outcomes between the adj TT and IO groups, nor exploring late recurrence patterns and their subsequent responses to IO or TT, as these may be different from the responses observed at early recurrence. Imaging follow‐up was performed every 3 months, however, the imaging modalities used varied. Moreover, the assessment of response to subsequent therapies was performed by investigators' discretion; thus, may have contributed to heterogeneity in the evaluation process.

## 5 CONCLUSION

Both adj TT and IO yielded prolonged RFS in a real‐world setting, consistent with previously published data from pivotal phase III trials that suggest similar efficacy between the two drug classes. However, longer follow‐up is necessary to determine the long‐term efficacy of these adj treatments in daily clinical practice, including any potential benefit in OS. Delineating long‐term OS benefit, or lack thereof, is particularly important given the high treatment‐related toxicity seen in this analysis. Overall, patients who are refractory and relapse during adj therapy constitute a group with poor outcomes, likely related to treatment resistant disease biology and need to be strongly considered for novel therapies and clinical trials. In contrast, patients who relapse after having discontinued adj therapy, particularly anti‐PD1 IO, appear to still benefit from IO re‐treatment. Adj TT may be less well tolerated than suggested in clinical trials, with lower rates of 1‐year completion in a real‐world setting. Larger studies are needed to clarify the optimal approaches to managing disease recurrence following adj therapy for stage III melanoma.

## AUTHOR CONTRIBUTIONS

**Danai Dima:** Conceptualization (equal); data curation (equal); formal analysis (equal); methodology (equal); writing – original draft (equal). **Nerea Lopetegui‐Lia:** Data curation (equal); project administration (equal); writing – review and editing (equal). **Olisaemeka Ogbue:** Data curation (supporting); software (equal). **Bennett Osantowski:** Data curation (supporting); writing – review and editing (equal). **Fauzia Ullah:** Data curation (supporting); software (equal); validation (equal); writing – review and editing (equal). **Xuefei Jia**: Formal analysis (supporting). **Jung Min Song:** Writing – review and editing (supporting). **Brian Gastman:** Writing – review and editing (supporting). **James Isaacs:** Supervision (supporting); writing – review and editing (supporting). **Lucy Boyce Kennedy:** Supervision (supporting); writing – review and editing (supporting). **Pauline Funchain:** Supervision (equal); validation (lead); writing – review and editing (lead).

## FUNDING INFORMATION

This research received no specific grant from any funding agency in the public, commercial, or not‐for‐profit sectors.

## CONFLICT OF INTEREST STATEMENT

Pauline Funchain: Consulting or Advisory Role (Eisai, Novartis, GigaGen), Research Funding (Pfizer, Bristol‐Myers Squibb, Taiho Oncology). Brian Gastman: Stock and Other Ownership Interests (Castle Biosciences), Consulting or Advisory Role (Castle Biosciences, Quest Imaging), Speakers' Bureau (Castle Biosciences), Research Funding (Alkermes, Instil Bio, Merck; NeoImmuneTech, Quest Imaging), Travel, Accommodations, Expenses (Alkermes). The rest of the authors have no conflict of interest to declare.

## Supporting information


Data S1.

## Data Availability

Due to privacy, ethical concerns and institution policy supporting data cannot be made openly available.

## References

[1] Robert C , Karaszewska B , Schachter J , et al. Improved overall survival in melanoma with combined dabrafenib and trametinib. N Engl J Med. 2015;372(1):30‐39. 25399551 10.1056/NEJMoa1412690

[2] Robert C , Schachter J , Long GV , et al. Pembrolizumab versus ipilimumab in advanced melanoma. N Engl J Med. 2015;372(26):2521‐2532. 25891173 10.1056/NEJMoa1503093

[3] Robert C , Long GV , Brady B , et al. Nivolumab in previously untreated melanoma without BRAF mutation. N Engl J Med. 2015;372(4):320‐330. 25399552 10.1056/NEJMoa1412082

[4] Robert C , Grob JJ , Stroyakovskiy D , et al. Five‐year outcomes with dabrafenib plus trametinib in metastatic melanoma. N Engl J Med. 2019;381(7):626‐636. 31166680 10.1056/NEJMoa1904059

[5] Dummer R , Ascierto PA , Gogas HJ , et al. Overall survival in patients with BRAF‐mutant melanoma receiving encorafenib plus binimetinib versus vemurafenib or encorafenib (COLUMBUS): a multicentre, open‐label, randomised, phase 3 trial. Lancet Oncol. 2018;19(10):1315‐1327. 30219628 10.1016/S1470-2045(18)30497-2

[6] Dummer R , Ascierto PA , Gogas HJ , et al. Encorafenib plus binimetinib versus vemurafenib or encorafenib in patients with BRAF‐mutant melanoma (COLUMBUS): a multicentre, open‐label, randomised phase 3 trial. Lancet Oncol. 2018;19(5):603‐615. 29573941 10.1016/S1470-2045(18)30142-6

[7] Larkin J , Chiarion‐Sileni V , Gonzalez R , et al. Combined nivolumab and ipilimumab or monotherapy in untreated melanoma. N Engl J Med. 2015;373(1):23‐34. 26027431 10.1056/NEJMoa1504030 PMC5698905

[8] Atkins MB , Lee SJ , Chmielowski B , et al. Combination dabrafenib and trametinib versus combination nivolumab and ipilimumab for patients with advanced BRAF‐mutant melanoma: the DREAMseq trial‐ECOG‐ACRIN EA6134. J Clin Oncol. 2023;41(2):186‐197. 36166727 10.1200/JCO.22.01763 PMC9839305

[9] Ascierto PA , Del Vecchio M , Mandalá M , et al. Adjuvant nivolumab versus ipilimumab in resected stage IIIB‐C and stage IV melanoma (Checkmate 238): 4‐year results from a multicentre, double‐blind, randomised, controlled, phase 3 trial. Lancet Oncol. 2020;21(11):1465‐1477. 32961119 10.1016/S1470-2045(20)30494-0

[10] Eggermont AMM , Blank CU , Mandalà M , et al. Adjuvant pembrolizumab versus placebo in resected stage III melanoma (EORTC 1325‐MG/KEYNOTE‐054): distant metastasis‐free survival results from a double‐blind, randomised, controlled, phase 3 trial. Lancet Oncol. 2021;22(5):643‐654. 33857412 10.1016/S1470-2045(21)00065-6

[11] Grossmann KF , Othus M , Patel SP , et al. Final analysis of overall survival (OS) and relapse‐free‐survival (RFS) in the intergroup S1404 phase III randomized trial comparing either high‐dose interferon (HDI) or ipilimumab to pembrolizumab in patients with high‐risk resected melanoma. J Clin Oncol. 2021;39(15_suppl):9501.

[12] Grossmann KF , Othus M , Patel SP , et al. Adjuvant pembrolizumab versus IFNα2b or ipilimumab in resected high‐risk melanoma. Cancer Discov. 2022;12(3):644‐653. 34764195 10.1158/2159-8290.CD-21-1141 PMC8904282

[13] Dummer R , Hauschild A , Santinami M , et al. Five‐year analysis of adjuvant dabrafenib plus trametinib in stage III melanoma. N Engl J Med. 2020;383(12):1139‐1148. 32877599 10.1056/NEJMoa2005493

[14] Eggermont AMM , Chiarion‐Sileni V , Grob JJ , et al. Adjuvant ipilimumab versus placebo after complete resection of high‐risk stage III melanoma (EORTC 18071): a randomised, double‐blind, phase 3 trial. Lancet Oncol. 2015;16(5):522‐530. 25840693 10.1016/S1470-2045(15)70122-1

[15] Tarhini AA , Lee SJ , Hodi FS , et al. Phase III study of adjuvant ipilimumab (3 or 10 mg/kg) versus high‐dose interferon alfa‐2b for resected high‐risk melanoma: north American intergroup E1609. J Clin Oncol. 2020;38(6):567‐575. 31880964 10.1200/JCO.19.01381 PMC7030886

[16] Weber JS , Schadendorf D , Del Vecchio M , et al. Adjuvant therapy of nivolumab combined with ipilimumab versus nivolumab alone in patients with resected stage IIIB‐D or stage IV melanoma (Checkmate 915). J Clin Oncol. 2023;41(3):517‐527. 36162037 10.1200/JCO.22.00533 PMC9870220

[17] Weber J , Mandala M , Del Vecchio M , et al. Adjuvant nivolumab versus ipilimumab in resected stage III or IV melanoma. N Engl J Med. 2017;377(19):1824‐1835. 28891423 10.1056/NEJMoa1709030

[18] Moyers JT , Chong EG , Mitchell J , Patel A , Jeong ISD , Nagaraj G . Abstract 4338: immunotherapy in resected stage III melanoma: an analysis of the National Cancer Database. Cancer Res. 2020;80(16_Supplement):4338.

[19] Zaremba A , Eggermont AMM , Robert C , et al. The concepts of rechallenge and retreatment with immune checkpoint blockade in melanoma patients. Eur J Cancer. 2021;155:268‐280. 34392069 10.1016/j.ejca.2021.07.002

[20] Ng G , Xu W , Atkinson V . Treatment approaches for melanomas that relapse after adjuvant or neoadjuvant therapy. Curr Oncol Rep. 2022;24(10):1273‐1280. 35639333 10.1007/s11912-022-01288-y PMC9474352

[21] Owen CN , Shoushtari AN , Chauhan D , et al. Management of early melanoma recurrence despite adjuvant anti‐PD‐1 antibody therapy☆. Ann Oncol. 2020;31(8):1075‐1082. 32387454 10.1016/j.annonc.2020.04.471 PMC9211001

[22] Bhave P , Pallan L , Long GV , et al. Melanoma recurrence patterns and management after adjuvant targeted therapy: a multicentre analysis. Br J Cancer. 2021;124(3):574‐580. 33087895 10.1038/s41416-020-01121-y PMC7851118

[23] Gershenwald JE , Scolyer RA , Hess KR , et al. Melanoma staging: evidence‐based changes in the American Joint Committee on cancer eighth edition cancer staging manual. CA Cancer J Clin. 2017;67(6):472‐492. 29028110 10.3322/caac.21409 PMC5978683

[24] Eggermont AMM , Kicinski M , Blank CU , et al. Association between immune‐related adverse events and recurrence‐free survival among patients with stage III melanoma randomized to receive pembrolizumab or placebo: a secondary analysis of a randomized clinical trial. JAMA Oncol. 2020;6(4):519‐527. 31895407 10.1001/jamaoncol.2019.5570 PMC6990933

[25] Long GV , Hauschild A , Santinami M , et al. Adjuvant dabrafenib plus trametinib in stage III BRAF‐mutated melanoma. N Engl J Med. 2017;377(19):1813‐1823. 28891408 10.1056/NEJMoa1708539

[26] De Falco V , Suarato G , Napolitano R , et al. Real‐world clinical outcome and safety of adjuvant therapy in stage III melanoma patients: data from two academic Italian institutions. Int J Cancer. 2023;153(1):133‐140. 36752579 10.1002/ijc.34462

[27] De Meza MM , Blokx WAM , Bonenkamp JJ , et al. Adjuvant BRAF‐MEK inhibitors versus anti PD‐1 therapy in stage III melanoma: a propensity‐matched outcome analysis. Cancer. 2023;15(2):409. 10.3390/cancers15020409 PMC9857200 36672358

[28] Eggermont AMM , Blank CU , Mandala M , et al. Adjuvant pembrolizumab versus placebo in resected stage III melanoma. N Engl J Med. 2018;378(19):1789‐1801. 29658430 10.1056/NEJMoa1802357

[29] Livingstone E , Forschner A , Hassel JC , et al. Multicenter real‐world data of adjuvant treatment and disease outcome of patients with melanoma with high‐risk of recurrence. J Clin Oncol. 2022;40(16_suppl):9570.

[30] de Meza MM , Ismail RK , Rauwerdink D , et al. Adjuvant treatment for melanoma in clinical practice—trial versus reality. Eur J Cancer. 2021;158:234‐245. 34600790 10.1016/j.ejca.2021.08.044

[31] van Laar SA , Kapiteijn E , Gombert K , Guchelaar HJ , Zwaveling J . 812P early experiences in adjuvant treatment of melanoma: real‐world data on tolerability, safety and efficacy. Ann Oncol. 2022;1(33):S917.

[32] Schreuer M , Jansen Y , Planken S , et al. Combination of dabrafenib plus trametinib for BRAF and MEK inhibitor pretreated patients with advanced BRAFV600‐mutant melanoma: an open‐label, single arm, dual‐centre, phase 2 clinical trial. Lancet Oncol. 2017;18(4):464‐472. 28268064 10.1016/S1470-2045(17)30171-7

[33] Valpione S , Carlino MS , Mangana J , et al. Rechallenge with BRAF‐directed treatment in metastatic melanoma: a multi‐institutional retrospective study. Eur J Cancer. 2018;91:116‐124. 29360604 10.1016/j.ejca.2017.12.007

[34] Cybulska‐Stopa B , Rogala P , Czarnecka AM , et al. BRAF and MEK inhibitors rechallenge as effective treatment for patients with metastatic melanoma. Melanoma Res. 2020;30(5):465‐471. 32221131 10.1097/CMR.0000000000000662

[35] Eggermont AMM , Kicinski M , Blank CU , et al. Five‐year analysis of adjuvant pembrolizumab or placebo in stage III melanoma. NEJM Evidence. 2022;1(11). doi:10.1056/EVIDoa2200214 38319852

[36] Olson DJ , Eroglu Z , Brockstein B , et al. Pembrolizumab plus ipilimumab following anti‐PD‐1/L1 failure in melanoma. J Clin Oncol. 2021;39(24):2647‐2655. 33945288 10.1200/JCO.21.00079 PMC8376314

[37] Betof Warner A , Palmer JS , Shoushtari AN , et al. Long‐term outcomes and responses to retreatment in patients with melanoma treated with PD‐1 blockade. J Clin Oncol. 2020;38(15):1655‐1663. 32053428 10.1200/JCO.19.01464 PMC7238490

[38] Dummer R , Gyorki DE , Hyngstrom J , et al. Neoadjuvant talimogene laherparepvec plus surgery versus surgery alone for resectable stage IIIB‐IVM1a melanoma: a randomized, open‐label, phase 2 trial. Nat Med. 2021;27(10):1789‐1796. 34608333 10.1038/s41591-021-01510-7

[39] Taylor AM , Galea C , Lo SN , et al. Efficacy and safety of “second adjuvant” therapy with BRAF/MEK inhibitors after resection of recurrent melanoma following adjuvant PD‐1–based immunotherapy. J Clin Oncol. 2022;40(16_suppl):9575. 10.1016/j.ejca.2024.113561 38278009

