# Supplementary material for: Real‐world outcomes of patients with resected stage III melanoma treated with adjuvant therapies
Source: Cancer Med. 2024 Jun 20;13(12):e7257. doi: 10.1002/cam4.7257 (PMC11190025; doi:10.1002/cam4.7257)
Supplement: Supplementary file 1 — Data S1. [file CAM4-13-e7257-s001.docx]

**Table S1.** Baseline characteristics of patients with BRAF mutation.

| **Clinical Characteristics** | **Targeted Therapy**  N=30 | **Immunotherapy**  N=42 |
| --- | --- | --- |
| **Age, median (range)** | 54 (33-80) | 54 (30-79) |
| **Male Gender – n(%)** | 15 (50) | 26 (62) |
| **White Race – n(%)** | 30 (100) | 42 (100) |
| **ECOG PS, median (range)** | 0 (0-2) | 0 (0-2) |
| **Stage III – n(%)**  A  B  C  D | 5 (17)  10 (33)  14 (47)  1 (3) | 7 (17)  16 (38)  18 (43)  1 (2) |
| **Primary Site – n(%)**  Head & Neck  Trunk  Lower Extremities  Upper Extremities | 6 (20)  11 (37)  5 (17)  8 (26) | 7 (17)  14 (33)  10 (24)  11 (26) |
| **Type of Lymph Node Involvement (for stage >IIIb disease, n=61) – n(%)**  Macroscopic  Microscopic | 0 (0)  15 (100) | 0 (0)  42 (100) |
| **KIT mutation – n(%)**  Positive  Negative  Not documented | 0 (0)  20 (67)  10 (33) | 1 (2)  28 (67)  13 (31) |
| **NRAS mutation – n(%)**  Positive  Negative  Not documented | 0 (0)  20 (67)  10 (33) | 1 (2)  28 (67)  13 (31) |


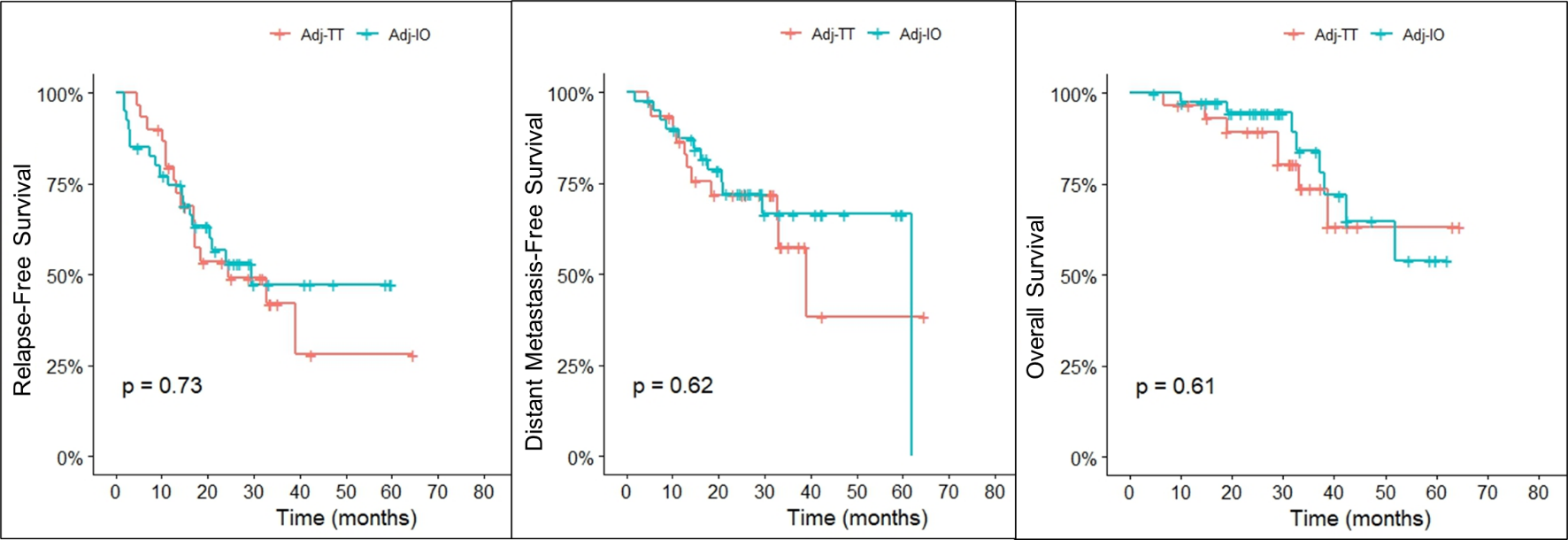
**Figure S1.** Kaplan-Meier Curve for Progression-Free Survival, Distant Metastasis-Free Survival and Overall Survival for patients with BRAF mutation.

**Table S2.** Patients who relapsed from the Adjuvant TT group: Patterns of Recurrence, Management and Outcomes (excluding those who received experimental therapies) (N=15)

| **Pattern**  **of Recurrence** | **Relapse ON**  **vs OFF Adj-Rx** | **1-year**  **Adj-Rx** | **Systemic Rx at relapse** | **Response** | **Further progression** | **Site** | **Status** |
| --- | --- | --- | --- | --- | --- | --- | --- |
| **Locoregional** | | | | | | | |
| P5 | OFF | Yes | Pembro | NED | No |  | Alive |
| P10 | ON | No | Nivo | NED | Yes | Local | Alive |
| P19 | OFF | Yes | Nivo | NED | No |  | Alive |
| P21 | OFF | No | Nivo | NED | Yes | Local | Dead |
| P23 | OFF | Yes | Nivo | NED | Yes | Local | Alive |
| **Distant** | | | | | | | |
| P1 – lung | OFF | No | Nivo | SD | No |  | Alive |
| P6 – lung | OFF | No | Nivo | PD | Death |  | Dead |
| P24 – spleen | OFF | No | Nivo | CR | No |  | Alive |
| P26 – lung | OFF | Yes | Nivo | PR | No |  | Alive |
| P9 – lung | OFF | No | Ipi/Nivo | SD | No |  | Alive |
| P14 – brain | ON | No | Ipi/Nivo | PD | Death |  | Dead |
| P17 – liver | OFF | No | Enco/Bini | PR | Yes | Liver | Dead |
| P25 – brain | OFF | Yes | Enco/Bini | SD | Yes | Brain | Dead |
| P27 – brain | ON | No | Darb/Tram | PD | Yes | Brain | Dead |
| P15 – brain, liver bone | OFF | Yes | None | PD | Death |  | Dead |

Abbreviations: Adj=adjuvant; Darb/Tram=dabrafenib/trametinib; CR=complete response; Enco/Bini= encorafenib/ binimetinib; Ipi/Nivo=ipilimumab/Nivolumab; NED=no evidence of disease; Nivo=nivolumab; P=patient; PD=progressive disease, Pembro= pembrolizumab; PR=partial response; Rx=treatment; SD=stable disease

**Table S3.** Patients who relapsed locoregionally from the Adjuvant IO group: Patterns of Recurrence, Management and Outcomes (excluding those who received experimental therapies) (N=17).

|  | **Relapse ON**  **vs OFF Adj-Rx** | **1-year**  **Adj-Rx** | **Systemic Rx at relapse** | **Local intervention** | **Response** | **Further progression** | **Site** | **Status** |
| --- | --- | --- | --- | --- | --- | --- | --- | --- |
| P32 | OFF | Yes | Nivo | resection | NED | No |  | Alive |
| P48 | OFF | Yes | Nivo | resection | NED | No |  | Alive |
| P65 | ON | No | Pembro | resection | NED | Yes | LN, bone | Alive |
| P110 | OFF | Yes | Pembro | resection | NED | No |  | Alive |
| P80 | ON | No | Ipi/Nivo | resection | NED | Yes | LN, bone | Alive |
| P107 | ON | No | Ipi/Nivo | resection | NED | Yes | Local | Alive |
| P116 | ON | No | Ipi/Nivo | resection | NED | Yes | LN | Alive |
| P61 | ON | No | Nivo | None | CR | No |  | Alive |
| P35 | OFF | Yes | Nivo | TVEC | PR | No |  | LTF |
| P45 | OFF | No | Pembro | TVEC | CR | No |  | Alive |
| P53 | OFF | Yes | Pembro | TVEC | CR | No |  | Alive |
| P77 | ON | No | Pembro | TVEC | PD | Yes | Bone, Lung, GI, Kidney | Dead |
| P82 | ON | No | Nivo | TVEC | PD | Yes | Local | Dead |
| P86 | ON | No | Pembro | TVEC | PR | Yes | Local | Alive |
| P40 | ON | No | Enco/Bini | resection | NED | Yes | Brain | Dead |
| P55 | ON | No | Darb/Tram | resection | NED | Yes | Local | Alive |
| P85 | ON | No | Darb/Tram | resection | NED | Yes | Local | Alive |

Abbreviations: Adj=adjuvant; Darb/Tram=dabrafenib/trametinib; Enco/Bini= encorafenib/ binimetinib; LTF=loss to follow up; Ipi/Nivo=ipilimumab/Nivolumab; NED=no evidence of disease. Nivo= nivolumab; P=patient; Pembro= pembrolizumab; Rx=treatment

**Table S4.** Patients who relapsed with distant metastasis from the Adjuvant IO group: Patterns of Recurrence, Management and Outcomes (excluding those who received experimental therapies) (N=18).

|  | **Site at first relapse** | **Relapse ON**  **vs OFF Adj-Rx** | **1-year**  **Adj-Rx** | **Systemic Rx at relapse** | **Response** | **Further progression** | **Site** | **Status** |
| --- | --- | --- | --- | --- | --- | --- | --- | --- |
| P34 | Lung | ON | No | Nivo | CR | No |  | Alive |
| P39 | Bone, LN | OFF | No | Nivo | SD | Yes | Bone, Parotid | Dead |
| P67 | Liver | OFF | No | Nivo | SD | No |  | Alive |
| P69 | Spleen | OFF | Yes | Nivo | CR | No |  | Alive |
| P79 | brain | ON | No | Nivo | SD | No |  | Alive |
| P84 | ST, LN, lung | OFF | Yes | Nivo | CR | No |  | Dead |
| P94 | Lung, Brain | OFF | Yes | Nivo | CR | No |  | Alive |
| P120 | Colon | OFF | Yes | Nivo | CR | No |  | Alive |
| P38 | Bone, Stomach, Peritoneum | OFF | Yes | Ipi/Nivo | PD | Yes/Death | Peritoneum, Liver | Dead |
| P51 | Bone, Lung,  ST, LN | ON | No | Ipi/Nivo | N/A | Death (pneumonia) |  | Dead |
| P59 | Liver, Lung, LN, Pancreas, Bone | OFF | Yes | Ipi/Nivo | SD | Death (sepsis) |  | Dead |
| P98 | Lung | ON | No | Ipi/Nivo | PR | No |  | Alive |
| P99 | Brain | ON | No | Ipi/Nivo | PD | Yes | Brain | Dead |
| P108 | Bone, LN | ON | No | Ipi/Nivo | SD | Yes | Brain | Dead |
| P42 | Brain, Liver  ST, LN | ON | No | Ipi | SD | Yes | Liver, LN, ST | Dead |
| P60 | Lung, LN, Brain | OFF | No | Enco/Bini | SD | Yes | Brain | Dead |
| P106 | Lung | OFF | Yes | Darb/Tram | CR | Yes | Brain | Dead |
| P89 | Liver, Small Bowel, LN | OFF | No | None | PD | Yes/Death | Liver, Small Bowel, LN | Dead |

Abbreviations: Adj=adjuvant; Darb/Tram=dabrafenib/trametinib; CR=complete response; Enco/Bini=encorafenib/ binimetinib; Ipi/Nivo=ipilimumab/Nivolumab; LN=lymph nodes; N/A=not assessed; Nivo=nivolumab; P=patient; PD=progressive disease, Pembro=pembrolizumab; PR=partial response; Rx=treatment; SD=stable disease,

ST=soft tissue.

**Table S5.** Best Overall Response to Systemic Therapy at First Relapse based on Timing of Relapse (ON vs OFF adjuvant therapy).

|  | Timing of relapse | First line of therapy | | | |
| --- | --- | --- | --- | --- | --- |
|  |  | TT | Anti-PD1 | ipi/nivo | ipi |
| Adj-TT | ON adj-TT | n=1  ORR=0%  (PD) | n=1  (NE)  - | n=1  ORR=0%  (PD) | 0  -  - |
|  | OFF adj-TT | n=2  ORR=0%  (1 SD, 1 PD) | n=8  ORR=50%  (1 CR, 1 PR, 1 SD, 1 PD, 4 NE) | n=1  ORR=0%  (SD) | 0  -  - |
| Adj-IO | ON adj-IO | n=3  (NE)  - | n=7  ORR=50%  (2 CR, 1 PR, 1 SD, 2 PD, 1 NE) | n=7  ORR=25%  (1 PR, 1 SD, 1 PD, 1 NA, 3 NE) | n=1  ORR=0%  (SD) |
|  | OFF adj-IO | n=2  ORR=100%  (1 CR, 1 PR) | n=12  ORR=78%  (6 CR, 1 PR, 2 SD, 3 NE) | n=2  ORR=0%  (1 SD, 1 PD) | 0  -  - |

Abbreviations: adj=adjuvant, CR=complete response, IO=immunotherapy, n=number of patients, NA=not assessed, NE=non-evaluable, ORR=overall response rate, PD=progressive disease, PR=partial response, Rx-therapy, SD=stable disease, TT=targeted therapy.

**Table S6.** Best Overall Response to Systemic Therapy at First Relapse based on Location of Relapse (locoregional vs distant).

|  | Location of relapse | First line of therapy | | | |
| --- | --- | --- | --- | --- | --- |
|  |  | TT | Anti-PD1 | ipi/nivo | ipi |
| Adj-TT | Locoregional | n=5  (NE) | 0  - | 0  - | 0  - |
|  | Distant | n=3  ORR=33%  (1 PR, 1 SD, 1 PD) | n=4  ORR=50%  (1 CR 1 PR, 1 SD, 1 PD) | n=2  ORR=0%  (1 SD, 1 PD) | 0  -  - |
| Adj-IO | Locoregional | 3  (NE)  - | n=11  ORR=71%  (3 CR, 2 PR, 2 SD, 4 NE) | 3  (NE)  - | 0  -  - |
|  | Distant | n=2  ORR=50%  (1 CR, 1 SD) | n=8  ORR=63%  (5 CR, 3 SD) | n=6  ORR=17%  (1 PR, 2 SD, 2 PD, 1 NA) | n=1  ORR=0%  (SD) |

Abbreviations: adj=adjuvant, CR=complete response, IO=immunotherapy, n=number of patients, NA=not assessed, NE=non-evaluable, ORR=overall response rate, PD=progressive disease, PR=partial response, SD=stable disease, TT=targeted therapy.
